# Public Health in East and Southeast Asia: Challenges and Opportunities in the Twenty-First Century

**DOI:** 10.3201/eid1908.130721

**Published:** 2013-08

**Authors:** Carol Ciesielski

**Affiliations:** Centers for Disease Control and Prevention (retired), Atlanta, Georgia, USA

**Keywords:** public health, Southeast Asia, East Asia

Writing a book about public health in East and Southeast Asia is a daunting challenge. Comprising Mongolia to the north and Indonesia to the south, with all the countries in between, East and Southeast Asia are home to >2 billion persons and include the full economic and development spectrum of nations in the 21st century. Because of this regional complexity, providing a cohesive, comprehensive overview of public health issues, which involves making generalizations while trying to provide the right level of detail and contrast, is an ambitious goal. However, it was not completely met by this text.

The book addresses such topics as the area’s changing societal norms and lifestyles, emerging infectious and chronic diseases, nutrition, tobacco use, injuries, occupational health, health services, and globalization. Its strengths include the chapters on chronic diseases, tobacco, and injuries, which provide a good general overview of these issues in the region, with a detailed look at mental health issues. The chapters on infectious diseases are sparse in detail, however, and lack in-depth discussions of the context that places Asia at such high risk for becoming the source of pandemics. 

A rather confusing organization places a description of the control of emerging and other communicable diseases in a separate section of the book (the health services section). However, besides a few redundancies in the chapters, thought-provoking discussions on economics are provided. Data from specific countries are presented somewhat randomly throughout the chapters, sometimes resulting in fragmented discussions.

This volume may be a useful addition to those studying public health issues in East and Southeast Asia, especially its sections on chronic diseases, injuries, and tobacco. Nonetheless, this book should be supplemented by more detailed texts for in-depth studies of individual countries or disease states.

